# A qualitative study of perceived needs and factors associated with the quality of care for common mental disorders in patients with chronic diseases: the perspective of primary care clinicians and patients

**DOI:** 10.1186/s12875-016-0531-y

**Published:** 2016-09-13

**Authors:** Pasquale Roberge, Catherine Hudon, Alan Pavilanis, Marie-Claude Beaulieu, Annie Benoit, Hélène Brouillet, Isabelle Boulianne, Anna De Pauw, Serge Frigon, Isabelle Gaboury, Martine Gaudreault, Ariane Girard, Marie Giroux, Élyse Grégoire, Line Langlois, Martin Lemieux, Christine Loignon, Alain Vanasse

**Affiliations:** 1 Department of Family Medicine and Emergency Medicine, Université de Sherbrooke, 3001, 12th Avenue North, Sherbrooke, QC Canada; 2 St. Mary’s Hospital Center, 3830 Lacombe Avenue, Montreal, QC Canada; 3 CISSS de la Montérégie-Est, 90 Sainte-Foy Boulevard, Longueuil, QC Canada; 4 Université de Sherbrooke, UMF Chicoutimi, 305, St-Vallier, Chicoutimi, QC Canada; 5 Université du Québec à Chicoutimi, 555, Boulevard de l’Université, Chicoutimi, QC Canada; 6 Université de Sherbrooke - Campus de la santé, Groupe de recherche PRIMUS, 3001, 12e avenue nord, Sherbrooke, QC J1H 5N4 Canada

**Keywords:** Anxiety disorders, Major depression, Chronic diseases, Primary care, Quality improvement, Qualitative research, Patient experience, Treatment

## Abstract

**Background:**

The prevalence of comorbid anxiety and depressive disorders is high among patients with chronic diseases in primary care, and is associated with increased morbidity and mortality rates. The detection and treatment of common mental disorders in patients with chronic diseases can be challenging in the primary care setting. This study aims to explore the perceived needs, barriers and facilitators for the delivery of mental health care for patients with coexisting common mental disorders and chronic diseases in primary care from the clinician and patient perspectives.

**Methods:**

In this qualitative descriptive study, we conducted semi-structured interviews with clinicians (family physician, nurse, psychologist, social worker; *n* = 18) and patients (*n* = 10) from three primary care clinics in Quebec, Canada. The themes explored included clinician factors (e.g., attitudes, perception of roles, collaboration, management of clinical priorities) and patient factors (e.g., needs, preferences, access to care, communication with health professionals) associated with the delivery of care. Qualitative data analysis was conducted based on an interactive cyclical process of data reduction, data display and conclusion drawing and verification.

**Results:**

Clinician interviews highlighted a number of needs, barriers and enablers in the provision of patient services, which related to inter-professional collaboration, access to psychotherapy, polypharmacy as well as communication and coordination of services within the primary care clinic and the local network. Two specific facilitators associated with optimal mental health care were the broadening of nurses’ functions in mental health care and the active integration of consulting psychiatrists. Patients corroborated the issues raised by the clinicians, particularly in the domains of whole-person care, service accessibility and care management.

**Conclusions:**

The results of this project will contribute to the development of quality improvement interventions to increase the uptake of organizational and clinical evidence-based practices for patients with chronic diseases and concurrent common mental disorders, in priority areas including collaborative care, access to psychotherapy and linkages with specialized mental health care.

**Electronic supplementary material:**

The online version of this article (doi:10.1186/s12875-016-0531-y) contains supplementary material, which is available to authorized users.

## Background

Anxiety and depressive disorders are the most frequent mental health problems in the general population [1, 2] and among patients with chronic diseases [3–6]. The reciprocal relationship between common mental disorders and chronic diseases is associated with consequences on disability, quality of life, individuals’ health state and mortality [6–9]. Low detection rates have been found for anxiety disorders [10] and depression [11, 12], and primary care studies generally report that less than one person out of two receives a minimally adequate pharmacological and psychological treatment based on clinical practice guidelines for anxiety or depression [13–16]. Findings of previous studies are inconsistent regarding the association between the presence of chronic conditions and the quality of primary mental health care [17, 18], which emphasizes the need for knowledge on the delivery of mental health services for depression and anxiety disorders in patients suffering from chronic physical diseases in primary care.

**Fig. 1:**
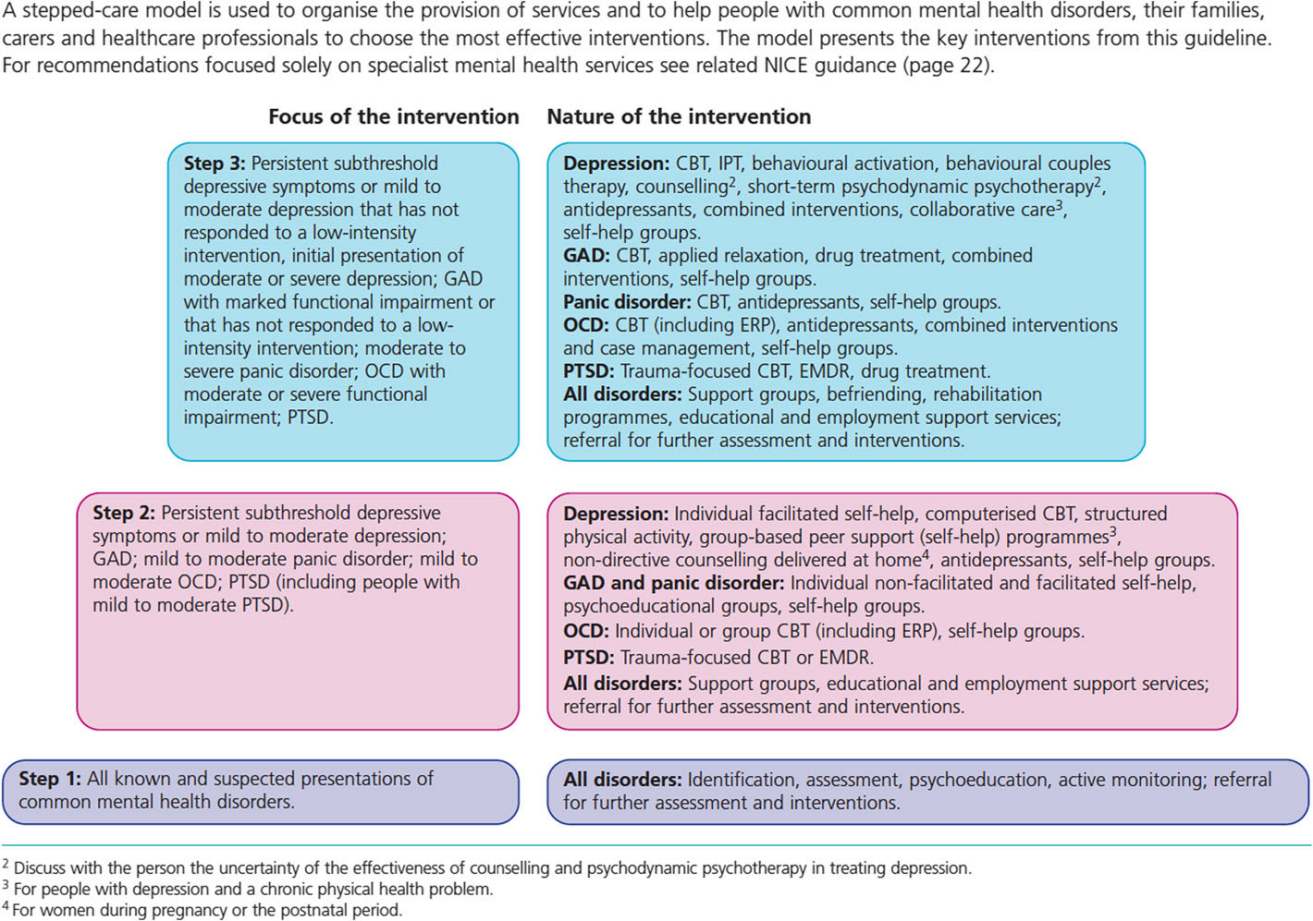
The stepped-care model. Source: NICE (2011). NICE clinical guideline 123. Quick reference guide.(p.6) [53]

Multifaceted clinical challenges are associated with the management of both common mental disorders and chronic diseases. Coexisting conditions can influence patient-clinician collaboration, and increase the complexity of diagnosis and treatment for primary care clinicians, particularly for the detection of mental disorders, the coordination of care, the prescription of complex medication regimens and competing priorities during time-limited consultations [19]. For patients, challenges for the management of both types of conditions may also be reflected by a high burden of treatment and limited self-management capacities [4]. In the United Kingdom, Coventry and colleagues [20] conducted a qualitative study in primary care with health professionals, patients and caregivers to explore the barriers associated with screening and management of depression in people with diabetes or coronary heart disease. Barriers to screening and treatment were present when, among other things, depression was seen as a normal response to chronic illness. The normalization of psychological distress in the presence of chronic diseases may lead to either under-detection by clinicians or under-reporting of depressive symptoms by patients [20, 21]. Coventry suggested that better communication between clinician and patient would permit the achievement of a shared understanding of the presence of depression and that a greater collaboration would enable a more adequate management of depression.

Knowledge production regarding the optimal management of depression and anxiety in patients with chronic diseases is rapidly evolving, but its uptake in service organization and clinical practices is much slower. Numerous studies show that improving the quality of care and the health status of patients in primary care is achievable by implementing complex interventions that include both organizational and educational components; some complex organizational interventions that target the structure and processes of care delivery have demonstrated their effects on both the care process and patients’ health status, and have often involved inter-professional collaboration [22–24]. In a perspective of integrating clinical and organizational practices to enhance the efficiency of mental health care, effective treatment of anxiety and depressive disorders in primary care should include complex strategies of collaborative care and of stepped care [25–27]. Collaborative care models include the support of a case manager and a mental health specialist, and have proven effective in improving the health of people with anxiety and depression [23, 28]. Furthermore, stepped care models are characterized by services which are modulated according to the severity of symptoms, take into account patient needs and preferences, and include the development of a systematic and continuous evaluation of patient’s response to treatment [27, 29].

Clinical research must turn towards the active involvement of primary care providers and patients to help guide further research orientations and the implementation of evidence-informed changes in primary mental health care practice. The implementation of these changes in clinical settings must use rigorous evidence as well as a proven integrated knowledge application approach that takes into account the potential users and the context in which knowledge is applied [30]. This project aimed to study the perception of clinicians and patients regarding the delivery of mental health services for depression and anxiety disorders in patients suffering from chronic physical diseases in primary care in the organizational context of the province of Quebec (Canada). Our study is based on the methodology used by Coventry and colleagues [20] to identify interventions most likely to improve clinical care and meet patients’ needs and preferences. In order to analyse the needs and subsequently propose adapted solutions to improve quality of care, the specific objectives were to explore the needs and challenges perceived by clinicians regarding the delivery of mental health care for patients with chronic diseases and concurrent common mental disorders, to examine the facilitators and barriers to the uptake of evidence-based-practices, and to study the needs and challenges perceived by patients regarding their mental health treatments in the context of the care they receive for their chronic disease in primary care.

## Methods

### Research design and study sites

We conducted a qualitative descriptive study in three primary care clinics in the province of Quebec (Canada). The three clinics were university-affiliated Family Medicine Units (FMU), which deliver health services to local populations and provide medical training to residents. The project was the start-up project of the University of Sherbrooke Department of Family Medicine and Emergency Medicine’s Practice Based Research Network (PBRN), one of the four primary care PBRNs of the Réseau-1 Québec, a network of primary health decision makers, care researchers, clinicians and patients which aims to generate and apply patient-oriented knowledge in priority health domains. The clinics were chosen according to diversity in terms of their local health network’s size, resources, and geographic environment. Local work committees were created in each clinic to validate the study protocol and to facilitate integrated knowledge transfer.

### Participants and recruitment

Clinicians and patients were recruited from each of the three clinics. The inclusion criteria for clinicians were: 1) provision of services to patients with chronic diseases; 2) at least 12 months of clinical experience. Inclusion criteria for patients were: 1) age 18 years or older, 2) presence of a chronic disease (e.g. diabetes, arthritis, chronic obstructive pulmonary disease); 3) depression or anxiety disorder (panic disorder, agoraphobia, social anxiety disorder or generalized anxiety disorder) in the past 2 years according to clinician’s diagnosis; 4) good knowledge of French or English; 5) having a family physician in one of the three clinics. Exclusion criteria for patients were the inability to provide consent, cognitive impairment, a history of manic episodes or a psychotic disorder.

A purposive sampling strategy was planned to recruit clinicians to ensure representation of each clinic, clinician type (family physician, nurse, psychologist, social worker) and gender. For patients, representation by gender, age, and socioeconomic status were sought.

The project was presented to clinicians in the three clinics by researchers (PR, CH) during team meetings in order to recruit clinicians and to present patient recruitment tools (waiting room advertisement and brochure). This recruitment strategy was adapted in each clinic in collaboration with the clinical managers and following consultation with the clinics’ local steering committees. Interested clinicians then gave their name to the researchers or contacted the research team directly by telephone or email to signal their interest in participating. Clinicians were also invited to give a project brochure to patients corresponding to inclusion criteria, and interested patients contacted the research team to participate. Patients were either identified and recruited directly by clinicians, or they self-referred after consulting the advertisement in the three clinic’s waiting rooms. Patients were not paired with their primary care providers in the recruitment approach. Upon the first contact, a research assistant verified participants’ eligibility and interview dates were scheduled.

### Data collection

Data collection tools included brief self-reported questionnaires and semi-structured interview guides (provided as Additional files 1 and 2) for clinicians and patients. The patient questionnaire included socio-demographic and medical history questions. The clinicians’ version included socio-demographic and professional practice questions. The research team developed semi-structured interview guides for patients and clinicians based on a stepped care model for common mental disorders [29], which was operationalized by including the principles of stepped care (Fig. 1) treatment delivery and monitoring as the main sections of the interview: detection and diagnosis, treatment choice according to severity of symptoms, low-intensity interventions (e.g. psychoeducation, self-help, group interventions), high-intensity interventions (e.g. antidepressants, cognitive behavior therapy, collaborative care), continuity of care, stratified and stepped approaches, and measurement-based care. We also built on the interview guides developed by Coventry and colleagues [20]. Themes explored in the clinician interviews included, for example: attitudes and knowledge, training and continuing education, perception of their role, current clinical practices, use of clinical practice guidelines, quality improvement barriers and facilitators, access to psychotherapy, inter-professional and inter-organization collaboration. Patient interviews included questions on access to care and services, needs and preferences, challenges of managing a chronic disease and depressive and/or anxiety symptoms, communication with health professionals. The interview guides were developed to provide the flexibility essential to allow the emergence of new themes. The project steering committee in each clinic validated the protocol and interview guides before starting the project. Two patient representatives also reviewed the interview guides to ensure that the themes covered in the interview guides were relevant to patients’ concerns.

A psychologist (HB) with experience as a knowledge broker in mental health services conducted all interviews. Participants were interviewed on an individual basis. Although we aimed at conducting all interviews with a face-to-face contact with respondents at the three primary care clinics, a secondary option was also to manage interviews by telephone on a case-by-case agreement due to geographical logistics and respondent’s preference. Interviews were conducted between March and August 2014 and were expected to last approximately 60 min, including the presentation of the project and signature of the informed consent form. The consent forms of participants who had a telephone interview were scanned or faxed to the research team.

### Data analysis

Interviews were recorded and transcribed verbatim and NVivo qualitative data analysis software (Version 10; QSR International) [31] was used for data management. Data coding and analysis was conducted based on Miles and Huberman’s cyclical process of data reduction, data display and conclusion drawing and verification [32]. The mixed codification strategy was based on themes selected following the literature review, priorities perceived by the interdisciplinary research team and variables emerging during data analysis. Under the supervision of the two main researchers (PR, CH), a research assistant (AB) conducted the initial data coding. The interviews of two clinicians and one patient were co-coded by the research assistant (AB) and the interviewer (HB) and a satisfactory inter-rater agreement was achieved. The codification of interviews was carried out continuously to allow for minor revisions to the interview guides. The themes and analyses were further discussed among research team members. The two main researchers (PR, psychologist and CH, family physician) and the research assistant (AB) initially read and analysed all transcripts individually and then engaged in in-depth discussions on each theme in order to share perspectives and come to a consensus. Data triangulation for clinics and the complete dataset was conducted to ensure the validity of results and to foster a better understanding of the issues [32].

## Results

### Study sites and participants

FMU-1, with two service points, was located in urban and semi-urban settings and provided services to over 12,000 enrolled patients. One of the service points was located in the same building as a Local Community Services Centre (CLSC). The clinical team was composed of 27 family physicians, three nurses, two nurse practitioners, two part-time psychologists (one for adults and one for children and adolescents), one social worker, one part-time pharmacist, one clinical coordinator and 37 family medicine residents. The clinic did not have access to an assigned consulting psychiatrist.

FMU-2 was located on the premises of a hospital in a semi-urban region and had over 10,000 patients enrolled. The clinical staff was composed of 13 full-time family physicians and 2.6 nurses (full-time equivalent), and 22 family medicine residents. The CLSC provided the services of a psychologist and a social worker one day per week but these services were located off-site. A full-time psychologist provided training to clinicians and residents and coaching to nurses. A consulting psychiatrist was available to assist family physicians in the diagnosis and treatment of psychiatric illnesses. Four quality improvement research projects involving the implementation of evidence-based practices for anxiety and depression [33], case management by nurses and self-management support for frequent users with chronic disease [34], chronic disease prevention and management services in primary care with primary care physician and nurse collaboration [35] and health equity for individuals living in poverty [36] had recently been carried out or were simultaneously underway.

FMU- 3 was situated in a medium-sized urban hospital, located in one of Canada’s most densely populated and ethnically diverse neighbourhoods. Patients came from a variety of districts served by diverse Health and Social Service Centres. The clinic had some 30 000 registered patients. The clinical team was composed of 45 staff physicians, eight nurses, two nurse practitioners, one clinical coordinator and 56 family medicine residents. A full-time psychologist provided training to residents. A consultant psychiatrist was available for weekly case discussions with the residents and access to psychiatric services was available through a service contract with the CLSC (service provided off site).

#### Clinicians

Eighteen clinicians, six from each clinic, participated in the study. The sample comprised ten family physicians, six nurses, one psychologist and one social worker. One half of the interviews were held in-person and the others were conducted by telephone. The majority of participants were women (83 %), nearly half were in the 30–39 age group and 56 % had more than 10 years of experience in their profession. Eleven worked 4 days or more (≥28 h were week) at the clinic. They reported that the proportion of their patients with a chronic physical disease and common mental disorders ranged between 3 and 40 %, and clinicians saw these patients on average 5.5 times (*SD* = 3.4) per year. Overall, clinicians reported that they felt at ease evaluating and treating anxiety and depressive disorders. Clinicians were also asked to rate the extent to which they had access to a consulting psychiatrist to support them, and results varied from low for FMU-1 and FMU-2, to moderately high for FMU-3. The majority of clinicians (*n* = 16) reported having the support of other sources of mental health services (e.g. interdisciplinary teams, collaborative care, designated mental health telephone service). Clinicians reported attending an average of 1.7 half-days of continuing education related to mental health in the past 12 months. While family physicians’ payment method was in general a mix of salary and fee-for-service, all other clinicians were salaried employees.

#### Patients

Ten patients (FMU-1, *n* = 3; FMU-2, *n* = 2; FMU-3, *n* = 5) took part in the individual interviews; five were women and five were 60 years old or older. Seven participants were married or living with a partner; seven had a high school degree or less; seven perceived their economic situation as sufficient or financially secure. Four participants were studying or working, while the others were not working (e.g. health reasons, keeping house, retired). Mental health problems reported by participants included depression (*n* = 8) and/or anxiety disorder (*n* = 5). As seen in Table 1, the most frequent chronic diseases (grouped by condition categories) were cardiovascular conditions and associated risk factors, and musculoskeletal and pulmonary conditions. Six participants reported experiencing chronic diseases in more than one category of conditions.

**Table 1:** Individual characteristics of patients (*n* = 10)

Code	FMU	Gender	Perception of income level	Reported mental health problem	Reported chronic conditions^a^
P01	1	Man	Sufficient	Anxiety; Depression	1
P02	3	Woman	Sufficient	Depression	1, 2
P03	3	Woman	Very poor	Anxiety; Depression	1, 2, 3, 4
P04	2	Woman	Financially secure	Anxiety	1, 2
P05	2	Woman	Financially secure	Depression	1, 5
P06	3	Man	Sufficient	Anxiety	1, 3, 4
P07	3	Woman	Very poor	Depression	4
P08	1	Man	Sufficient	Anxiety; Depression	4
P09	3	Man	Poor	Depression	3
P10	1	Man	Financially secure	Depression	1,2

^a^1 = Cardiovascular condition or risk factor; 2 = Musculoskeletal condition; 3 = Gastrointestinal condition; 4 = Pulmonary condition; 5 = Urological condition

The following sections provide the results from the interviews with the clinicians and patients. The themes reported from the clinician interviews comprise: detection and diagnosis of depression or anxiety disorders, treatment (pharmacotherapy and psychotherapy), collaboration with other clinicians and resources, and the barriers and facilitators, which influence the optimal delivery of care. The analysis of the themes from the patient interviews are embedded in the clinician interview’s main themes.

### Detection and diagnosis of anxiety or depressive disorders

Challenges were reported by family physicians regarding the detection and diagnosis of common mental disorders. Clinicians concurred that greater attention is given to the physical symptoms and the management of the chronic disease than to mental health during consultations. Lack of time and similarity of somatic symptoms with chronic diseases (e.g. fatigue) were the main explanations provided. However, family physicians reported that a mental health assessment was included in the patients’ annual check-up. They also reported that patients vary in their willingness to discuss their anxiety or depressive symptoms, and when patients do not report symptoms overtly, clinicians must probe further, pay attention to covert signals. Long-term patient-clinician relationships facilitated the detection of depression or anxiety disorders as clinicians come to know their patients well in addition to occasionally knowing other family members who may also report their relative’s mood variations during their own consultations.

*«(…) after a number of years, we are more in the nuances, so I would say yes, the subtle, indirect, clues (…) I always say ‘welcome to the wonderful world of the gray zone’, that’s what medicine is (…)» (C12_FMU-1_MD)*

Many patients perceived their family physician had important time constraints and hesitated to take some time to discuss their mental health. A conflicting result among patients was that, while some patients appreciated that their care providers considered their chronic disease and their depression or anxiety disorder in conjunction, other patients preferred that the two conditions be considered separately, and only the condition motivating the consultation be addressed.

*« She doesn’t just see me as the sick person. Her attitude is that she treats the whole person, not just the symptoms. I feel comfortable saying to her, listen I’m having a bad day today. » (P07_FMU-3_woman)*

*«I don’t want to bring those two together. If they’re feeling sick they’re there for that reason. If they’re feeling mentally sick they go to the other doctor. But when you go for a physical from the doctor for your physical health, it should only be about that.» (P03_FMU-3_woman)*

Some patients with depression or anxiety disorders reported feeling stigmatized by care providers, which caused anxiety and could influence their disclosure of symptoms and treatment choice.

*«We as mental health patients or people with psychiatric problems do not like to be on medication because there’s always a stigma attached to it. Because people hear you’re sick and that’s all they see. » (P07_FMU-3_woman)*

Clinicians did not frequently use screening tools for depression or anxiety disorders, except by nurses in FMU-2 where depression and anxiety self-reported assessment scales (e.g. Patient Health Questionnaire-9 [37], Generalized Anxiety Disorder-7 [38]) were included in their evaluation toolkit.

### Pharmacotherapy

The standard treatment option for anxiety and depressive disorders in the clinics was pharmacotherapy. Family physicians and nurses reported that adherence to pharmacological treatments was a challenge for patients with chronic diseases and common mental disorders. These patients required regular follow-up and increased attention to patient education, in particular for patients who were resistant to adding another medication to their already complex medication regimen. Thus, the assessment of patient readiness was influential, and the therapeutic relationship between patients and clinicians facilitated patient acceptance and adherence to pharmacotherapy. Clinicians frequently cited polypharmacy as a challenge in treatment of complex patients, whether at the level of the management of medications’ side effects, drug interactions or patient treatment adherence, and also mentioned the need for further training on psychopharmacotherapy.

*«(…) when we have more information, sometimes it’s easier to see the connection between the possible side effects, with how the patient can react, so I find that we are missing a bit of training.» (C13_FMU-2_nurse)*

*«(…) if we have more rounds about psychiatry and about different pharmacological options, and perhaps more people with chronic diseases, that would be a help.» (C14_FMU-3_ MD)*

### Psychosocial and psychological interventions

Clinicians reported employing a diversity of low-intensity interventions including supportive psychotherapy, lifestyle education, self-help support, motivational interviewing and basic cognitive-behavioural therapy interventions. They felt competent in this role, and were clear about their own professional boundaries in the provision of high-intensity interventions such as cognitive-behavioural therapy. Nurses were more invested than physicians in low-intensity interventions, possibly due to their scope of practice. In FMU-2, the provision by nurses of patient self-management support, which includes behavioural activation, was being implemented and this engagement was reflected in their discourse. Group therapy was not available in the clinics but some clinicians referred to community resources for specific group interventions (e.g. a self-management group based on the Stanford program and a group for the treatment of anxiety disorders).

From the patient standpoint, respondents usually indicated an appreciation for the involvement of care providers other than family physicians. They particularly commented on the care they receive from nurses and highlighted their complementarity and their availability.

*«Yes, she’s a nurse practitioner. (…). Sometimes, we could talk about other things than the disease; it was helpful too.» (P02_FMU-3_woman)*

While provisional psychological support was available from a psychologist or social worker in FMU-1 and FMU-2, these services were insufficient in terms of availability. Clinicians from the three clinics stressed the importance of improving access to psychotherapy and their aspiration to offer such services on their premises. Patient factors related to clinicians’ decision making regarding referral to psychotherapy included their preferences, interests, motivation, level of insight, socioeconomic status, and access to an employee assistance program or insurance plan covering psychotherapy. Some clinicians reported helping their patients clarify their goals they wish to attain before referring them to a psychologist in order to better prepare them for psychotherapy and to help them benefit more from their therapy. Patients were not generally directed to a specific type of therapy. However, some clinicians did acknowledge that cognitive-behavioural therapy was known to be effective for anxiety disorders. Some clinicians felt that brief solution-oriented therapies were more appropriate for their patients.

### Interprofessional collaboration & access to community resources

As mentioned in the description of the three clinics, health care professionals working with family physicians in the clinics varied, and the degree of involvement in mental health care of these professionals also differed across settings. For instance, FMU-1 did not allocate a specific role for nurses in mental health follow-up, whereas in FMU-2 nurses’ role in the area of mental health was highly developed and their leadership was confirmed and consolidated through research projects underway. These nurses were involved in the systematic follow-up of patients with mental disorders, in the assessment of their level of functioning, and the referral to other services such as psychotherapy. They were supported in their functions by an easy access to family physicians and the support and mentoring of a psychologist.

The availability and involvement of psychiatrists varied across clinics and the perception of their accessibility also varied among clinicians within a same clinic. Whereas a recent organizational agreement was expected to provide an access to a consulting psychiatrist in FMU-1, access to the consulting psychiatrist was difficult for the patients served by FMU-2. In FMU-3, clinicians did not have access to a consulting psychiatrist and contacted the psychiatrist on duty at the general hospital if required.

Targeted, short-term consultations for the evaluation of medications, in particular for patients taking several medications and at risk of interactions, were reported as difficult to obtain if the patient’s condition was not urgent.

*«(…) if the patient needs a consultation in psychiatry, well it can take 6 months before he sees a psychiatrist and he won’t be well during these 6 months, he will just worsen (…)» (C04_FMU-2_MD)*

*« If I have a doubt about a diagnosis, the psychiatrist will not see my patient if he is not in a crisis.» (C01_FMU-1_MD)*

*«(…) we do have a consulting psychiatrist, he’s not here every day of the week and every evening for instance, so it is sometimes rather difficult (…)» (C04_FMU-2_MD)*

Access to pharmacists also differed across the clinics. FMU-1 had a pharmacist with an expertise in mental health on-site 2 days per week, while the other two clinics contacted pharmacists from the hospital’s pharmacy department or local pharmacies when needed. When a pharmacist was not present on site, observed differences in levels of collaboration between clinicians and pharmacists appeared to be attributable to clinicians’ own professional networks.

*«We are fortunate to have a pharmacist here who can not only help us in our selection of the best medication according to interactions, but who can also keep track of medication adjustments based on symptoms and all that…» (C11_FMU-1_MD)*

The clinical staff of FMU-1 included a social worker, a resource valued by the clinic’s professionals. The social worker revealed that colleagues’ perception of his role and competencies changed over time, evolving from that of offering guidance and support for financial difficulties towards that of a professional familiar with community resources, able to provide short-term psychosocial support for patients while they are on the waiting list for psychosocial services and able to have an eye open for detecting mental health problems.

*«(…) I have a problem with such or such a patient, then he meets them and suggests a community resource to them, it’s really very helpful. Because honestly, we don’t have the time to do all of that.» (C03_FMU-1_MD)*

Although brief interventions were available from a psychologist in FMU-1 and FMU-2, clinicians generally mentioned experiencing difficulties collaborating with psychologists. Clinicians who referred patients to private practice psychologists found it difficult to identify specific therapists whom they perceived as trustworthy and suggested patients consult the provincial psychologists corporation’s website to find one. On the other hand, a psychologist suggested that better knowledge concerning the benefits of psychotherapy could be helpful for clinicians when proposing therapy and could subsequently increase patients’ willingness to engage in psychotherapy. Many clinicians deplored lack of communication with psychologists and mentioned that consultation reports were very rarely provided. Despite these challenges, increased collaboration with psychologists was seen as a priority to enhance care effectiveness as their expertise and the complementary of their role was acknowledged. Finally, some clinicians suggested that psychologists could benefit from increased training on the nature and treatment of chronic diseases to enhance their understanding of patients’ medical care.

*«Yes, I am certain that psychologists are trained to know the physical symptoms of mental illness, but as (…) certain symptoms may be caused by one or the other, it’s very difficult, so working together, I think it might be more useful. »(C05_FMU-3_ MD)*

Patients expressed that they would appreciate to involve psychologists in their care and those who consulted a psychologist reported that it had a positive effect on their wellbeing.

*« I don’t really receive therapy as much as I’d like to. Because I’d like to be able to talk to somebody just to have a sounding board to be able to talk.» (P07_FMU-3_woman)*

*«Yes, it helped me a lot to understand myself, to open up (…)» (P06_FMU-3_man)*

The knowledge and referral to existing community resources in FMU-2 was recently facilitated by meetings with the community organizations and by the creation of a database of the services provided by these resources. In FMU-3, clinicians varied regarding their knowledge and use of community resources. Whereas the large territory served by FMU-3 made it difficult to be familiar with the substantial number of services scattered throughout the city, the smaller territory covered by FMU-2 eased familiarity with the more limited number of resources.

### Barriers and facilitators to optimal care delivery

The main perceived barriers affecting optimal mental health care included the limited availability of mental health services, the burden of care, delays accessing specialized services, suboptimal inter-professional communication and collaboration, as well as training needs. Patient’s burden of care (e.g. frequent consultations, multiple treatments) influenced clinicians’ and patients’ readiness to add other services such as psychotherapy. Clinicians clearly expressed the barriers limiting the accessibility of psychotherapy and psychiatry. Clinicians referred patients to private practice psychologists when they had private or collective insurance for complementary health services or access to an Employee Assistance Program. They referred the other patients who could not assume the cost of psychotherapy to their Health and Social Services Center, but access to services was characterized by long waiting lists, complex pathways, multiple practitioners and often unspecialized services.

*«Often we have a patient in front of us, for example, and if you want to communicate with the CLSC [local community service centre], if you want to contact their social worker, it’s just long, it’s difficult, I find the hours are problematic, it is not open on weekends (…). Access is very difficult and then, after that, when patients see a psychotherapist, see a social worker, receive the support, I never know, I do not know what is happening, I have to wait to see the patient to see what happened. It’s communication, it’s access, yes.» (C16_UMF-3_MD)*

Participants also exposed different barriers influencing the access the health services such as unavailability, cost, compatibility and perceived unavailability. Referral to external services by health professionals was occasionally inadequate due to language issues or due to care providers’ lack of knowledge of existing resources.

*« When I said I want to see a therapist she handed me a paper with different places I can go to try to get help. Half of them are in French, so they won’t take me. (…) And sometimes it costs money (…). If you’re on welfare, welfare’s not going to pay for it.». (P07_FMU-3_woman)*

*«We don’t really talk about mental health so how can people have access to a therapist when they don’t even know where to go (…). I meet doctors and often I ask them ‘Do you have resources in your community’, and the doctors don’t know.» (P09_FMU-3_man)*

Clinicians also reported difficulty in gaining access to psychiatrists in non-urgent situations. Finally, clinicians reported that beyond formal education, continuing education in mental health was essential in developing their competence and maintaining their knowledge up to date.

The prevailing facilitating factors and proposed solutions pointed to strategies to increase and ease collaboration. An essential ingredient was the interest in providing collaborative care. The use of shared tools and records, simplified care pathways and communication mechanisms were seen as means to ease collaboration and foster continuity of care. The presence of other health professionals on teams (such as pharmacists, psychologists and social workers) was highly valued. A participant clearly identified the importance of working in an interdisciplinary approach as critical to collaborative care:

*«(…) we don’t work in silos, and having this collaboration then leads us to become self-sufficient too, because we learn, because we develop mechanisms together, there is a community of practice, it’s all give and take, so I think the more we collaborate and the more we learn from each other (…).» (C11_FMU-1_MD)*

Overall, the information provided by the patients complemented the data gathered during the clinicians’ interviews. Some patients suggested that their mental illness brought a stigmatisation that influenced their willingness to disclose symptoms, affected health care professionals’ approach and the care they received, but overall, patients were satisfied with their experience of care. The issues concerning the care attributes reported by patients corroborated the topics raised by the clinicians, particularly in the areas of whole-person care, service accessibility and care management. Concerning their mental health care, some patients indicated that communication and collaboration among services and clinicians were effective.

*« Doctors communicate among themselves very well, very rapidly, and I am impressed by the way it works here (..)» (P06_FMU-3_man)*

## Discussion

The main purpose of our study was to document clinicians’ and patients’ perceptions and experience regarding the delivery of mental health services for anxiety and depressive disorders in patients suffering from chronic diseases. Studies that take into account both clinicians’ and patients’ experiences are essential in order to clearly assess the barriers and facilitators that have an impact on the uptake of evidence-based practices [30, 39]. Consistent with previous literature [20, 40], the results of this project indicate that primary care mental health care for patients with chronic diseases involves challenges for detection, diagnosis and treatment. The participants reported several barriers affecting the quality of care and various solutions were set forth. Many of the barriers and solutions mentioned by clinicians were corroborated by patients’ views concerning, for example, the involvement of other health professionals in their care and access to psychotherapy.

Primary care patients face a number of barriers in access to care and treatment adequacy for common mental disorders, such as low rates of detection, access to evidence-based psychotherapy or sub-optimal pharmacological care. In the presence of chronic diseases, a particular challenge is associated with the comorbidity of mental disorders and physical health problems. Mental health care is essential to comprehensive patient care and may have an important influence on the management of the chronic disease and patients’ engagement in their care [6, 8, 41]. Among the factors associated with detection and diagnosis of mental health problems, both clinicians and patients remarked on the limited consultation time with family physicians that had an impact on the attention given to the detection of mental disorders. Furthermore, the physical symptoms of chronic diseases, which warranted clinicians’ attention, limited their availability to address other issues. Some patients also reported a misattribution from health professionals of physical symptoms of their chronic disease or other illness to their mental disorder. This phenomenon, known as ‘diagnostic overshadowing’, is associated with lowered or delayed help-seeking, under-reporting of symptoms, diminished treatment adherence, under-diagnoses and decreased adequacy of care [42]. This phenomenon may be related to certain patients’ desire to keep their chronic condition and mental disorder separate in their consultations. Patients’ desire to maintain their mental health care distinct was also identified in a large-scale pragmatic trial conducted in the UK [43]. Stigmatisation of mental disorders may also prevent patients from reporting symptoms to clinician. Normalisation of depression in the context of chronic diseases and hesitation to label a patient by diagnosing a mental disorder have been identified in previous studies as barriers to the detection of mental disorders [20].

Regarding the treatment of anxiety and depressive disorders, pharmacotherapy was the most frequent treatment proposed for depression and anxiety disorders, which is understandable given that patients generally consult a family physician for mental health problems [44, 45]. Support from psychiatrists and pharmacists was sought, but access to these professionals varied widely across clinics and clinicians. The need for additional training on psychoactive medications was emphasized by family physicians. Challenges in polypharmacy for patients with complex conditions were often mentioned by family physicians, which is not surprising as the use of multiple medicines is the norm in patients with chronic diseases. To compound the problem, there is often a lack of explicit guidance with regards to multimorbidity in clinical practice guidelines [46]. Prescribing the most appropriate evidence-based psychotropic medication in primary care is also associated with particular challenges, including the risk of adverse drug events and lack of adherence [47]. Pharmacists are highly-skilled in that domain, and predictably, the importance of collaborating with pharmacists was underscored by clinicians. This is an area for quality improvement that could be investigated further to better define the role of pharmacists within multidisciplinary primary care teams and in the community for patients with mental disorders (e.g. feedback to clinicians, promoting medication adherence) [48]. While the role of psychiatrists as mental health specialists within collaborative care models is well defined, the role of pharmacists within multidisciplinary primary care teams for patients with mental disorders have received less attention.

Low-intensity psychosocial and psychological interventions were common practice, and the importance of psycho-education was frequently set forth, although educational material was rarely made available. Clinicians generally acknowledged the contribution of psychotherapy in the treatment of depression or anxiety disorders, yet identified major barriers including its accessibility (e.g. costs, waiting lists), collaboration with psychologists, and patients’ willingness to engage in psychotherapy. In the context of our study, clinicians consistently voiced the lack of accessibility to psychotherapy as a barrier to offering this treatment option, and patients who did receive psychotherapy expressed their satisfaction or their desire to receive additional therapy. The access to psychotherapy is currently a challenge in the public system [15, 49] and the reimbursement of psychotherapy services is highlighted as an avenue to expand access to psychotherapy services [50]. Patients’ preferences and readiness to introduce and adhere to psychological or pharmacological treatments for their anxiety or depression need to be closely assessed as these elements are essential to treatment initiation and treatment engagement. The results of a recent meta-analysis of 34 studies [51] reveal that patients with common mental disorders were more likely to prefer psychotherapy over pharmacotherapy. According to clinical practice guidelines and stepped care models [2, 29], psychotherapy is a key component of evidence-based service delivery for common mental disorders. The inclusion of psychologists in clinics was reported as valuable in many regards. The presence of a psychologist and on-site access was a positive asset in FMU-1 and the presence of a psychologist in FMU-2 proved to be an important resource regarding mental health training. The benefits of the involvement of psychologists in primary care were also reported in previous studies [52], where family physicians reported that the integration of psychologists improved their work conditions, quality of life, patient care and their knowledge on the management of mental health problems, while patients reported improved quality of life and confidence in their ability to manage day-to-day problems. The implementation of a stepped care model with evidence-based psychotherapy for common mental disorders in the province of Quebec would require significant changes in the current mental health service delivery, as neither universal access to psychotherapy or treatment with evidence-based psychotherapy (e.g. cognitive-behavior therapy, interpersonal therapy) are currently implemented in primary care.

Clinical practice guidelines [2, 53] emphasize the importance of modulating treatment according to patient needs and preferences, but also based on the severity of symptoms, as depicted in stepped care models. The development of a systematic and continuous evaluation of patient’s response to treatment [27, 29] could be facilitated by the increased use of assessment tools such as the Patient Health Questionnaire [37] or the Generalized Anxiety Disorder Questionnaire [38] for the detection and follow-up of depressive and anxiety symptoms. While FMU-1 and FMU-3 clinicians rarely used such tools, the FMU-2 nurses had received training to use assessment material from the psychologist, as well as through peer training and expertise sharing. Nurses had access to a toolkit containing validated assessment tools and their use facilitated patients’ follow-up and communication with family physicians. Considering that the use of assessment tools was implemented with success in FMU-2 by nurses, that shared tools were noted as a key strategy to facilitate collaborative care by clinicians, and that routine symptom measurement is a critical component of stepped care models, other primary care clinics and types of providers in our provincial primary care context would benefit from implementing this relatively simple practice in a timely manner to improve the quality of care for anxiety and depression. While measurement-based care is underutilized in community-based mental health care, the benefits of this practice to optimize care are well established for common mental disorders [54, 55].

Overall, the fostering of initiatives to increase inter-professional collaboration and to increase access to care were seen as means to increase quality of care for patients. Results of this study show heterogeneity in inter-professional and inter-organizational collaboration between clinics, but also among professionals within clinics, suggesting that the implementation of a formal collaborative framework could help advance collaborative practices to improve care for patients. Collaborative care for depression and anxiety disorders is an efficient treatment approach and has been associated with greater treatment adherence and improved patient outcomes [23, 28]. Furthermore, results of a recent meta-analysis indicate that collaborative care practices improve both depression outcomes and quality of life of patients suffering from medical conditions [56]. Health professionals who participated in the present study consistently emphasized that inter-professional collaboration was central to improving quality of care and that involving a greater diversity of clinicians (e.g. pharmacists, psychologists, nurses, social workers) was beneficial to the quality of care. Results reflected the diversity of the three FMUs’ contexts, as seen in the different resources available, team composition, the professionals’ roles and their level of involvement in mental health care. The three FMUs developed interesting collaborative mental health practices and could learn from each other both at the level of optimal team composition and the allocation of professional roles. For instance, in FMU-1, the inclusion of an on-site pharmacist was an effective resource for physicians needing to review their choice of medication and possible interactions. Furthermore, the integration of the consulting psychiatrist role in clinics, a new function set forth in mental health ministerial policies in Quebec [57], was currently underway, which partially explains why perceptions on the availability of consulting psychiatrists varied within clinics and clinicians. In clinics, the consulting psychiatrists’ role will be to support family physicians and community mental health teams in the management of patients with more complex disorders through a variety of strategies (e.g. on-site consultations with patients or clinicians, dedicated telephone line). This financial and organizational initiative offers an opportunity to implement the collaborative care model in primary care, considering that the underpinning of this complex intervention relies on collaboration between general practitioners and mental health specialists. However, the successful implementation of the model in routine care would require a well-planned quality improvement program at the provincial level and active physician engagement with the organizational and professional components of the model [58]. We can expect that some barriers to implementation of the model would be similar to previous studies in other contexts, such as the lack of knowledge of clinicians about the collaborative care model, the need to adapt clinical information systems in each setting to facilitate communication, and lack of resources to support implementation [59]. An implementation pilot study in naturalistic conditions building on the Normalisation Process Model for complex interventions in health care, such as Knowles et al. [58] in the UK [60], would be highly valuable to further our knowledge on barriers and facilitators associated with the implementation of the model [58, 61].

### Study limits & strengths

The following set of methodological issues should be considered in the interpretation of the findings. Selection bias may have influenced the results, as participants’ characteristics, such as a specific interest in mental health or quality improvement, may be associated with the decision to participate in the study. Furthermore, the sampling strategy initially planned could not be completed due to recruitment difficulties, but we recruited diverse professionals from the clinics in order to collect a variety of perspectives and add depth to our data. To minimize social desirability bias in participants’ responses, the interviewer came from an outside organisation. To reduce the risk of reflecting research team’s subjectivity in the selection and formulation of interview questions, all of the research team, members of local steering committees and two patients were consulted in the preparation of interview guides. Similarly, structured data analysis involved data coding inter-rater agreement, comprehensive case discussions, and involvement of stakeholders in the interpretation of results. Data saturation for clinicians was not optimal for certain topics as participants came from diverse professional backgrounds and possessed various levels of engagement in mental health. Furthermore, data content reflected the participants’ profession in each clinic. Finally, the chosen patient recruitment approach was not optimal and narrowing selection criteria (e.g. selecting a limited number of chronic diseases) could yield more circumscribed results.

## Conclusion

Patients with chronic diseases and depression and/or anxiety disorders are common in primary care clinicians’ daily practice. Various needs and challenges emerged from the interviews from clinicians who identified different avenues to enhance the efficiency and quality of the care they provide. Main suggestions included facilitating communication and collaboration among primary care professionals, increasing access to psychotherapy, and easing interactions with specialized services. Patients corroborated many of the barriers and solutions mentioned by clinicians. Among particular challenges to address with mental health care for patients with chronic diseases, this study highlighted comprehensiveness of care as well as polypharmacy. Regarding the implementation of evidence based stepped care models, access to psychotherapy in our provincial context would require considerable efforts on the financial, organizational and professional levels. Further studies are required to examine the implementation of the components of a stepped care model embedding collaborative care for patients with chronic diseases. Inter-professional collaboration is highly valued by professionals and patients, and there is a need for further studies examining the implementation of the expanded psychiatrist role as well as the contribution of allied health professionals in primary care clinics, with an evidence-based practice perspective. This project has allowed to gain a better understanding of the needs and care experience of patients, as well as to obtain the perspective of clinicians concerning quality improvement opportunities. Lessons learned from this project will provide valuable information for the development and implementation of tailored strategies to support the uptake of evidence-based mental health practices and improve the quality of the care experience of people living with chronic diseases.

## Additional files

Additional file 1: Interview guide for clinicians. (DOCX 54 kb) Additional file 2: Interview guide for patients. (DOCX 55 kb)

## Abbreviations

CLSC: Local Community Services Centre
FMU: Family medicine units
PBRN: Practice based research network
