# Erratum to: A qualitative study of perceived needs and factors associated with the quality of care for common mental disorders in patients with chronic diseases: the perspective of primary care clinicians and patients

**DOI:** 10.1186/s12875-016-0564-2

**Published:** 2016-12-30

**Authors:** Pasquale Roberge, Catherine Hudon, Alan Pavilanis, Marie-Claude Beaulieu, Annie Benoit, Hélène Brouillet, Isabelle Boulianne, Anna De Pauw, Serge Frigon, Isabelle Gaboury, Martine Gaudreault, Ariane Girard, Marie Giroux, Élyse Grégoire, Line Langlois, Martin Lemieux, Christine Loignon, Alain Vanasse

**Affiliations:** 1 Department of Family Medicine and Emergency Medicine, Université de Sherbrooke, 3001, 12th Avenue North, Sherbrooke, QC Canada; 2 St. Mary’s Hospital Center, 3830 Lacombe Avenue, Montreal, QC Canada; 3 CISSS de la Montérégie-Est, 90 Sainte-Foy Boulevard, Longueuil, QC Canada; 4 Université de Sherbrooke, UMF Chicoutimi, 305, St-Vallier, Chicoutimi, QC Canada; 5 Université du Québec à Chicoutimi, 555, Boulevard de l’Université, Chicoutimi, QC Canada; 6 Université de Sherbrooke - Campus de la santé, Groupe de recherche PRIMUS, 3001, 12e avenue nord, Sherbrooke, QC J1H 5N4 Canada

## Erratum

In our article [1] we omitted to include the credit line for Figure 1 in the legend. The credit line is as follows: “Reproduced with permission from National Institute for Health and Care Excellence (formerly the National Institute for Health and Clinical Excellence) (2011) CG123 Common mental health problems: identification and pathways to care. Manchester: NICE. Available from https://www.nice.org.uk/guidance/cg123”.
