# Supplementary material for: A qualitative study of perceived needs and factors associated with the quality of care for common mental disorders in patients with chronic diseases: the perspective of primary care clinicians and patients
Source: BMC Fam Pract. 2016 Sep 13;17(1):134. doi: 10.1186/s12875-016-0531-y (PMC5020556; doi:10.1186/s12875-016-0531-y)
Supplement: Additional file 1: — Interview guide for clinicians. (DOCX 54 kb) [file 12875_2016_531_MOESM1_ESM.docx]

Interview guide for clinicians

(adapted from Coventry et al., 2011)

**1. Current role at the FMU:**

- Your responsibilities and functions;
- Involvement with patients with chronic diseases (CD) and mental health (MH) problems, including depression and anxiety disorders;

**2. According to your experience, in people with CD, MH problems…**

- Frequent in your clinical practice?
- Anxiety and depression: are they the most frequent MH problems?
- Does their prevalence vary in function of the CD or other patient characteristics?

**3. Detection of depressive and anxiety disorders in patients with CD**

- Specific challenges?
- Challenges related to the attribution / differentiation / symptom perception – related to the CD or anxiety disorder;
  - - - *Example: Fatigue is a common symptom of CD and depression*
- Symptoms reported by patient or only when asked?
- Routine screening for anxiety / depression in patients with CD: Where? When? How? By whom**?**
- Use of questionnaires or screening tools;

**4. Diagnosis:**

- Tools or procedures for the assessment of mental disorders?
- Additional challenges in patients with CD?
- Ability to make a specific diagnosis of depression or an anxiety disorder?
- Referrals for further assessment? If so, to whom?
- Access to resources, if needed? Consulting psychiatrist or other?
- How could things be improved? (Barriers and facilitators)

**5. Patient education concerning mental disorders**

- Information / tools or documents on the disorder, its evolution, the treatments available?
- Resources available? If yes, which ones?
- Areas for improvement? Available or missing resources in your community?

**6. Treatment of depression and anxiety disorders in patients with CD -** **general points/issues**

- Care and services usually offered for an anxiety disorder / depression
- Special challenges in providing care? Different in patients with CD?
- Main criteria that guide the choice of treatment (pharmacological, psychological or other)
- Using clinical practice guidelines (CPG) and other clinical decision aid tools
- Role of the patient (and family) in decision-making - how?

**7. Pharmacological treatment**

- Main criteria governing the choice of pharmacological treatment (e.g., preference, severity, drug interactions)
- Main criteria that guide the modification of treatment (e.g. tools - which ones and how / if not - would these help?)
- Collaboration with other professionals:
  - Collaboration with other professionals (e.g., nurses, psychiatry, pharmacy)
  - Where? FMU, CSSSS, specialized services?
  - How? Collaboration mechanisms (e.g., referral, follow-up and communication with other professionals)
  - At the FMU or other sites?
- Specific issues associated with the use of a pharmacological treatment
- Ways of improving the quality of care

**8. Psychotherapy**

- Main criteria governing the choice of a psychotherapy
- Main criteria governing the choice of a type of psychotherapy (e.g., cognitive-behavioral therapy, interpersonal psychotherapy, support, problem solving)?
- Offered alone or in a combined treatment (pharmacotherapy and psychotherapy)
- Do you get involved in the provision of psychotherapy: do you offer it, do you provide referrals, etc.
- Is psychotherapy an available treatment option in your network?
- Do you have any collaboration agreements with partners in your network to facilitate access?
- Specific issues related to psychotherapy
  - Collaboration with other professionals (e.g., psychology, psychiatry)
  - Where? FMU, CSSSS, specialized services?
  - How? Collaboration mechanisms (e.g., referral, follow-up with other professionals)
    - *If yes, what specific types of psychotherapy are available in your network?
    - What are the challenges related to…
- Solutions or ways of improving the access to psychotherapy?

**9. Other types of interventions (*low intensity*)**

- Bibliotherapy (books, websites, self-care guides)?
- Support for self-care/self-management?
- Other low intensity psychotherapies (e.g., motivational interviewing, behavioral activation, problem solving)

Physical exercises

***Note to interviewer*** : *for these items, according to the situation, attempt to go further and to develop the answer provided*. **If Yes**, are there patient profiles for whom these treatments are particularly aimed at / who respond well to these interventions? **If you do not offer these interventions**, do you think that these interventions are: Important? / Relevant? / Are there barriers or factors that facilitate the access and use of these interventions?

**10. Complementary questions concerning treatment**

- Do you have any special arrangements to ensure the follow up and continuity of care of patients with CD and mental disorders? (e.g., follow up and coordination with other professionals)
- What do you do for patients who have a mild disorder or refuse to start treatment? How do you follow up on their symptoms?
- Are there any specific issues related to continuity of care? Areas for improvement?
- **Crisis and Emergency -** Do you have access to services for patients who are at immediate or imminent danger to themselves or others because of their mental state (crisis, high suicidal risk) and require rapid treatment? *(specific agreements, accessibility, promptness of intervention)*

**11. Organisation of service and clinical practices – Collaboration**

*Explain the difference between resources (CSSS, community resources) and care providers (psychologist, consulting psychiatrist)*

- Are there contexts where you feel overwhelmed and you need support for these patients?
- What are the resources that help you the most in your clinical practice to better meet the mental health needs of patients who suffer from depression / anxiety and CD? How do these resources help you?
- Are there complementary or additional resources that may help you better meet the needs of your patients?
- What kind of collaboration would you like to have?
- How could they help you?

**12. Training and professional development**

- Do you feel well equipped to work with this clientele?
- What has been helpful in your training for the management of patient care?
- Specific training needs?

**13. In conclusion...**

- In general, what are the main barriers you encounter in providing care to this clientele?
- Are there other topics that you would like to discuss or information that you consider important and which could guide us in our work which aims to better understand your situation and your needs when treating patients with CD and depression and/or anxiety disorders?

**Reference for adaptation**

Coventry PA, Hays R, Dickens C, et al. Talking about depression: a qualitative study of barriers to managing depression in people with long term conditions in primary care. *BMC Family Practice*. 2011; 12:10.
