# Supplementary material for: A qualitative study of perceived needs and factors associated with the quality of care for common mental disorders in patients with chronic diseases: the perspective of primary care clinicians and patients
Source: BMC Fam Pract. 2016 Sep 13;17(1):134. doi: 10.1186/s12875-016-0531-y (PMC5020556; doi:10.1186/s12875-016-0531-y)
Supplement: Additional file 2: — Interview guide for patients. (DOCX 55 kb) [file 12875_2016_531_MOESM2_ESM.docx]

Interview guide for patients

(adapted from Coventry et al., 2011)

1. **Experience with long-term health conditions**
   1. **Can you tell me briefly:**

- how do you live with your chronic condition?
- how did your long-term health condition begin?
- can you tell me about your treatment and follow up?
  1. **In the past two years,** **could you briefly tell me about the care you have received for your long-term health condition?**
- health care services
- persons / professionals / other care providers
- the clinic(s) where you had your consultations
- frequency of medical visits and duration
- usually on appointment / frequent walk-in visits
  1. **Health care experience: Tell me briefly about your experience and your satisfaction with the care you have received for your long-term health condition:**
- **Access to care:** What are the strengths and weaknesses of the care and services you received? Have you ever struggled to get help for your anxiety or depressive symptoms (e.g., access, efficiency)? If so, what problems have you had and how did you cope or deal with them?
- **Efficiency:** To help you manage your anxiety or depressive symptoms, were there some treatments or services that worked better than others?
- **Patient** **preferences** discussed or taken into account.
- **Opinions** on the treatments / care / services received.
- **Shared decision making**: Do you think you have contributed and participated in decisions about your treatments / care? Were significant others involved (if desired)?
- **Communication**: Did health care professionals take the time to listen to you? Did you experience difficulties communicating with health care professionals? If yes, what could be the reasons?

1. **Anxiety and depressive symptoms**
   1. **Experience: You have already mentioned that there was a time when you felt anxious or depressed in the past two years. I'd like you to tell me about it.**

- What led you to talk about your symptoms? (*To whom did you talk about it?)*
- Did a health professional tell you that may be suffering from an anxiety or depressive disorder?
- Do you think that there is a link between your long-term health condition and feeling anxious or depressed?
- Living with a long-term health condition (coping): Many people find it emotionally difficult to adapt and live with a long-term health condition - have you ever struggled at this level? (e.g., excessive worry leading to sleep disturbance, negativity/pessimism, hopelessness, feeling overwhelmed)
- Before this long-term health condition, did you have problems with anxiety or depression?
  1. **Treatment of depression and anxiety disorders**
- Tell me about a memorable experience surrounding the care you have received for your depression or anxiety disorder? (positive or negative)
- For which problem did you receive this care (anxiety or depression)?
- Can you describe the main characteristics of the care you received? For example:
  - What type of treatment did you receive? Who provided the care? Where did you receive this care? At what moment did you receive the care and how long did it last?
- Do you feel that having a long-term condition influences the way you receive care and help for your emotional problems?
- Before having a long-term condition, did you ever receive a treatment for an emotional problem? Did you notice differences between the care you received for your depression and/or anxiety disorder?
  1. **Health care experience**

***Tell me briefly about your experience and your satisfaction with the care you have received for your emotional problems.***

- **Access to care:** What are the strengths and weaknesses of the care and services you received? Have you ever struggled to get help for your anxiety or depressive symptoms (e.g., access, efficiency)? If so, what problems have you had and how did you cope or deal with them?
- **Efficiency:** To help you manage your anxiety or depressive symptoms, were there some treatments or services that worked better than others?
- **Patient** **preferences** / **Opinions** on the treatments / care / services received
- **Shared decision making**: Do you think that you have contributed and participated in decisions about your treatment / care?
  1. **Access to evidence-based treatments: Research shows that some treatments have been proven to be effective to treat anxiety or depression. We are interested in whether you have access to the following services and, if necessary, to know your opinion on the treatments:**
- Drugs / psychotherapy (e.g. CBT) / support groups / support for self-care
- Did the health professionals whom you consulted talk to you about it?
- Has it been offered to you? Have you used this service? Difficulties or barriers to access it?
- If this service / treatment had been proposed to you, do you think you would have used it?
- *For psychotherapy:* Who? Where (clinic, private)? Who referred you? Was it easily accessible? Do you know the therapeutic approach?
  1. **Other types of treatment or more informal support (informal caregiver, alternative therapy):**
- Have you had access to other types of treatment?
- Are there other treatments you would have liked to have access to, both for your long-term condition and your anxiety / depressive symptoms?
- Openness of professionals towards alternative approaches?
  1. **Communication:**
- In general, do you feel that you can talk about your emotional problems, and with whom do you talk about these (e.g., your family/friends/MD/other health professionals/other)?
- Do your healthcare professionals ask you how you cope or feel regarding your mental health? If so, how do you feel to be asked or questioned about it?
- Problems: Have you ever struggled to communicate with health professionals regarding how you cope with emotional problems?
- In your experience, would you say that health professionals focus more on managing the physical symptoms of your long-term health condition or on how you cope with it?
- In addition to the support you receive for your long-term health condition, is there room to also express yourself on mental health?
  1. **Coordination of care:**
- Do you feel health professionals work together / in collaboration to help you?
- Is there room for improvement in the way professionals work together / coordinate your care?

1. **In conclusion…**

- Are there items related to your health care experience that I have not addressed and that you find important to mention?
- Would you have any suggestions that could improve the health care experience for people living with a long-term health condition who may need help dealing/coping with their anxiety or depressive symptoms?

**Reference for adaptation**

Coventry PA, Hays R, Dickens C, et al. Talking about depression: a qualitative study of barriers to managing depression in people with long term conditions in primary care. *BMC Family Practice*. 2011; 12:10.
